# Autophagy in cancer-associated fibroblasts: its role in gastrointestinal cancers

**DOI:** 10.1186/s12943-026-02626-5

**Published:** 2026-03-04

**Authors:** Vaishnavi Kuppala, Hasini Chintapudi, Gopalaiah Kovuru, Ganji Purnachandra Nagaraju, Bassel F. El-Rayes

**Affiliations:** 1 Division of Hematology and Oncology, University of Alabama at Birmingham, Birmingham, AL 35233 USA; 2 Northwest Guilford High School, 5240 NW School Road, Greensboro, NC 27409 USA; 3 Department of Chemistry, University of Delhi, Delhi, 110007 India; 4 Division of Medical Oncology, Moores Cancer Center, UC San Diego, La Jolla, San Diego, CA 92093 USA

**Keywords:** Gastrointestinal cancers, tumor microenvironment, cancer-associated fibroblasts, Autophagy, tumor progression, resistance, therapy

## Abstract

Gastrointestinal (GI) cancers are a heterogeneous group of cancers with high morbidity and mortality rates. Despite advancements in early detection markers and therapy, the outcome remains poor due to high tumor heterogeneity and metastasis. The microenvironment (ME) in GI cancers is highly dynamic and enriched with immune and endothelial cells, extracellular matrix (ECM), and stromal components, including cancer associated fibroblasts (CAFs), which play an essential role in tumor progression. CAFs modulate tumor progression through extensive crosstalk with cancer cells, immune cells, and ECM remodeling. CAFs secrete cytokines, growth factors, and exosomes that modulate tumor progression, epithelial to mesenchymal transition, angiogenesis, immune tolerance, and therapy resistance. By remodeling the ECM, CAFs create a protumorgenic microenvironment through the activation of key signaling pathways such as YAP/TAZ and integrin/FAK. CAFs are also heterogeneous, with diverse subsets exhibiting context-dependent pro- or anti-tumor function, as discussed in this review. Autophagy is a process that removes damaged cellular components to maintain homeostasis. In the early stage of cancer, autophagy suppresses tumor progression. Still, in the later stages of tumors, CAFs support survival, metabolic alterations, therapy resistance, and immune evasion, as such are regulated by signals like TGF-β/IL-6/ROS and pathways including AMPK/STAT3/NF-κB/SMAD and mTOR. The review discusses CAFs and modulation of autophagy in GI cancer progression, metastasis, and resistance to therapy. CAFs remodel the ME and induce immune tolerance, while autophagy in both cancer cells and CAFs allows survival under stress. By showing these interconnected mechanisms, the review identifies CAF autophagy as a critical therapeutic target to overcome resistance in GI cancers.

**Graphical Abstract:**

**Figure:**
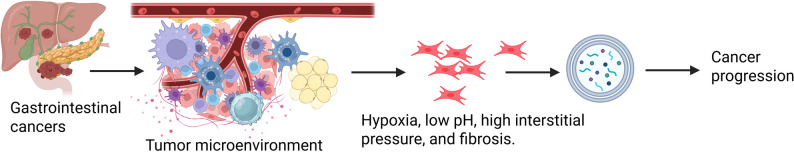
In gastrointestinal (GI) cancers, the microenvironment (ME) is rich in cancer associated fibroblasts (CAF) that secrete growth factors, which activate autophagy to provide energy for growing cells. Activated autophagy promotes tumor progression.

## Background

Gastrointestinal (GI) cancers constitute a heterogeneous group influenced by both adaptable and non-adaptable risk factors that determine progression to malignancy. In 2025, GI cancers incidence is approximately 362,200 new cases with 117,590 deaths USA [1]. Future predictions suggest that there will be over 30,000 new cases of esophageal cancer, 40,000 of gastric cancer, and 200,000 of CRC [1–3]. Colorectal cancer (CRC) proceeds in a stepwise manner, initially limited to the intestinal mucosa and submucosa, and, with lymphatic dissemination, then penetrates the deeper layers. 90% of CRCs are adenocarcinoma and spread via local invasion, lymphatic, and hematogenous routes to metastatic sites of the liver and lungs [4]. Delayed diagnosis is due to the lack of initial asymptomatic symptoms; diagnosis is made through the presence of CEA and CA19–9 tumor markers, endoscopy with biopsy, and imaging [5]. Treatment strategies include surgery, chemotherapy, radiotherapy, targeted therapy, and immunotherapies [6]. CRC outcomes depend on stage; early detection via colonoscopy is preferred, with a better prognosis, while prevention through diet, lifestyle, and regular screening remains crucial [7]. Pancreatic ductal adenocarcinoma (PDAC) is an aggressive cancer with a poor survival rate of only 10% in 5 years and is usually diagnosed at advanced III–IV stages [8]. PDAC is categorized as a precursor lesion, including PanINs, IPMNs, and MCNs, providing an opportunity for early detection [9]. Advances like IMMray PanCan-d, and a proteomic multi-biomarker assay (CA19-9) are highly sensitive and specific in the early stage [10]. Stomach cancer remains a global concern with a high death rate in eastern countries and men with a higher incidence rate [11]. However, long-term trends signify deteriorating incidence and mortality rates due to improved lifestyle and preventive measures, including reduced risk factors such as *H.pylori* infection, smoking, alcohol, and poor diet [12]. Esophageal cancer results due to genetic, environmental, and lifestyle factors, along with a precursor, Barrett’s esophagus (BE), which is caused by persistent gastroesophageal reflux disease leading to metaplastic transformation of normal squamous cells to adenocarcinoma [13]. Poor diet and lifestyle, along with tylosis, mutations in TP53, CDKN2A, and RB1, and NOTCH signaling alterations, increase the incidence rate [14]. Esophageal cancer treatment is stage-dependent, with endoscopic resection and ablation serving as a therapeutic option while surgical esophagectomy remains the standard for stage I disease in combination with chemoradiotherapy [15]. Challenges in GI cancer treatment include tumor heterogeneity and the complexity of the tumor microenvironment. Intratumoral heterogeneity leads to variable responses to standard therapies, along with unpredictable genetic profiling, microsatellite instability, and tumor cell composition [16]. High therapeutic resistance is predisposed by the TME niche, which promotes immune tolerance, stromal interactions, and angiogenesis, which limit the effectiveness of targeted treatments.

### Tumor microenvironment 

TME in GI cancers is a complex and heterogeneous, rich in cells, including cancer-associated fibroblasts (CAFs), immunocytes, blood vessels, ECM, and microvesicles [17]. The tumor microenvironment (TME) is featured by low oxygen, acidic pH, high interstitial pressure, and fibrosis that foster tumor progression and resistance to therapy [18]. The TME also contains tumor suppressor immune cells like natural killer cells, CD8^+^ cytotoxic T cells, M1 macrophages, Th1 cells, and pro-tumoral populations, including TAMs, MDSCs, Tregs, and neutrophils that promote immune evasion, neo vasculature, and metastasis [19]. Abnormal tumor vasculature causes poor drug delivery, while immune checkpoint PD-1/PD-L1 and CTLA4, expressed in the TME niche, cause immune tolerance [20]. CAFs are central regulators of therapy resistance in TME. In GI malignancies, CAF cells associate with tumor cells through the integrin- Focal Adhesion Kinase - Rho-associated coiled-coil kinase a mechano-transduction signaling axis and transcriptional regulators hippo pathway YAP/TAZ, promoting stiff ECM, fibrosis, and EMT, increasing tumor dissemination, and resistance to therapy [21]. CAF cells secrete platelet-derived growth factor-B; heparin-binding epidermal growth factor; and stromal-derived factor-1 /CXCL12, and tumor-derived PDGF-D and TGF-β1 show positive feedback activation of CAFs, promoting therapy resistance [22]. Thus, interactions among tumor cells, CAFs, ECM, immune constituents, and the vasculature hinder the efficacy of therapies.

### Autophagy in cellular balance and cancer

Autophagy is a cellular process that supports homeostasis by degrading and clearing intracellular components via lysosomal pathways. The stages of autophagy include the formation of phagophores, which transform into double-layered autophagosomes, which consume impaired organelles, misfolded protein aggregates, and even intracellular pathogens, followed by fusion with lysosomes for degradation [23, 24]. The degraded products are reutilized to sustain energy generation, macromolecular synthesis, and anaplerotic pathways under stress conditions. Autophagy can also function in substrate-specific receptors, including p62/SQSTM1, NBR1, and NDP52, that recognize polyubiquitinated cargos and recruit to LC3-marked autophagosomes and cause mitophagy, pexophagy, lipophagy, ferritinophagy, and xenophagy to maintain hemostasis [25]. Autophagy is essential for stress adaptation and is dysregulated, contributing to multiple diseases, including cancer. In cancer, autophagy has a dual role: it suppresses tumors by removing damaged organelles and oncogenic aggregates; however, it also facilitates tumor growth and metabolic adaptation [26, 27].

Cancer cells escape senescence by reactivating telomerase and promoting uncontrolled growth. With a decline in DNA repair factors, such as APE1/Ref-1, mutation accumulation increases, and ROS amplify DNA damage, activate HIF-1 and VEGF, and induce SASP via NF-κB, reinforcing inflammation and tumor progression [28]. Autophagy alleviates ROS-associated damage by removing dysfunctional mitochondria [29, 30]. Epigenetic alterations are hallmarks of cancer, along with chromatin remodeling and changes in DNA methylation. CpG islands are hypermethylated, and global hypomethylation disrupts the genome stability [31]. Sirtuins SIRT1/6 are known to regulate chromatin, DNA repair, and telomere maintenance, and also control EMT and metastasis [32]. Autophagy interacts with epigenetic regulators [33] and hinders mTOR, AMPK/LKB1, and PI3K to prevent the onset of neoplasia. Additionally, gut microbiota dysbiosis induces chronic inflammation, reduces tumor immune surveillance, and activates autophagy [34]. Thus, hereditary instability, epigenetic dysregulation, altered autophagy signal, and microbiota-driven inflammation together galvanize cancer progression and metastasis.

**Fig. 1:**
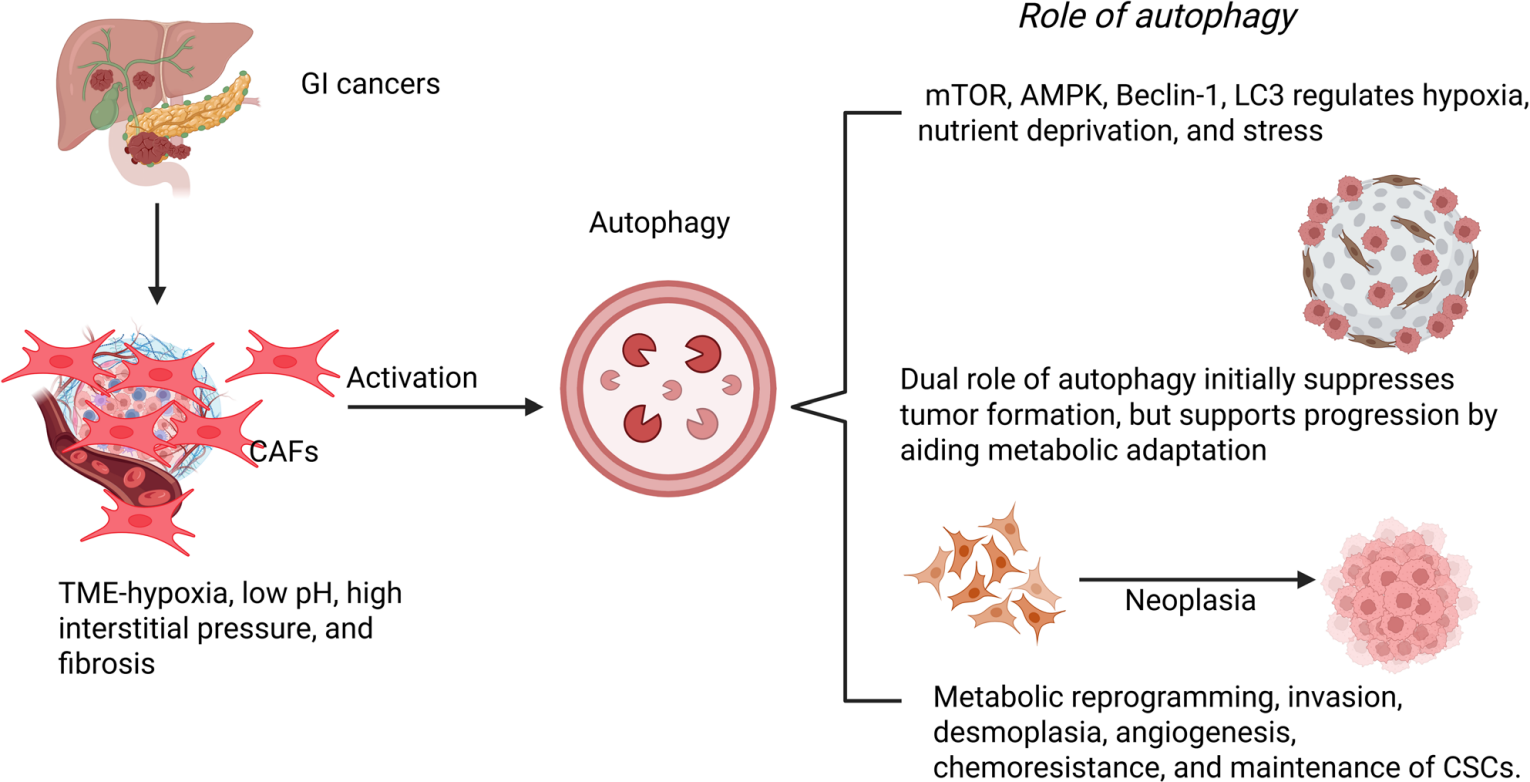
Cancer associated fibroblasts (CAFs)-autophagy crosstalk in gastrointestinal (GI) cancers. The tumor microenvironment (TME) in GI malignancies is characterized by hypoxia, low pH, and fibrosis, which activate CAFs. The activated CAFs undergo autophagy regulated by Beclin-, mTOR, AMPK, and LC3 under metabolic stress and nutrient deprivation. Autophagy plays a dual role in the initial stage of neoplasia: it suppresses tumor formation by maintaining cellular homeostasis, but later it promotes tumor progression through metabolic adaptation and facilitates metabolic reprogramming

CAFs display functional heterogeneity, with protumorigenic functions that dynamically crosstalk with cancer cells via cytokines, chemokines, and growth factors, thereby driving tumor progression and immune alterations [35]. Autophagy, regulated by mTOR, AMPK, Beclin-1, and LC3, plays a dual role in cancer: as a cytoprotectant, a metabolic regulator, and a stress-survival mechanism, but can also mediate cytotoxicity in a context-dependent manner [36]. In CAFs, oxygen and nutrition deprivation induce autophagy, triggering metabolic reprogramming [37]. CAF-autophagy crosstalk promotes GI malignancies through invasion, chemoresistance, desmoplasia, angiogenesis, and therapy resistance by maintaining CSCs and EMT, and by reducing sensitivity to chemotherapy and immunotherapy (Fig. 1).

### CAFs in the tumor microenvironment

The stromal component, including multipotent mesenchymal progenitors, pericytes, adipocytes, and CAFs, shapes tumor behavior by secreting cytokines, chemokines, fatty acids, and growth factors that promote angiogenesis, EMT, immune tolerance, and metabolic rewiring [38]. CAFs are activated fibroblasts within the TME, characterized by markers such as α-SMA, PDGFRα/β, FSP1, and FAP; however, no single universal biomarker exists, with FAP being the most clinically relevant due to its selective expression and tumor-promoting role [39]. CAFs, which developed as the dominant coordinators of TME crosstalk, influencing ECM remodeling, fibrosis, desmoplasia, and an immune-suppressive niche, also exhibit context-dependent tumor-restraining subtypes [40]. This web of networks at the intercellular level demonstrates that tumor development is not exclusively driven by cancer cells but is mechanistically embedded in reciprocal signaling loops between malignant and stromal populations [41]. CAFs originate from multiple sources, including resident fibroblasts, PSC/HSC stellate cells, MSCs, bone marrow-derived cells, EMT/EndMT epithelial or endothelial cells, MMT macrophages, and even pericytes or adipocytes [42], demonstrating lineage plasticity. The transformation from normal fibroblasts to CAFs is regulated by transcription and epigenetic rewiring of PRRX1 and SOX2 stimulation [42], DNA methylation, H3K27ac, H3K4me1 histone remodeling, and metabolic enzymes such as NNMT [43], while TGF-β, IL-6, IL-1, TNF-α, LIF, soluble factors, and hypoxia-driven HIF-1 signaling support activation [43]. miR-149, miR-27a, and miR-200 also fine-tune this phenotype [44]. Functionally, CAFs exhibit marked heterogeneity revealed by scRNA seq, subsets as cytokine-secreting- iCAFs; contractile α-SMA^+^, myCAFs apCAFs [45]; MHC-II plus immune modulators; ECM remodeler eCAFs and CAF-S clusters therapy-resistant, with each inhabiting separate spatial niches and functional states [46]. All these subsets play protumorigenic roles by stimulating proliferation, angiogenesis, immunosuppression, ECM remodeling, and therapy resistance, yet, inconsistently, a few CAF subtypes, including collagen-generated myCAFs, restrain tumor spread and support immune surveillance.

In the majority of GI cancers, the TME is extremely immunosuppressive, with immune cell collapse and persistent inflammation, thereby limiting active antitumor responses. Studies performed by scRNA and spatial transcriptomics demonstrated that T cell collapse specifically CD8^+^ intratumoral lymphocytes, which associate with aggressiveness, metastasis, poor response to therapy, and cancer progression [47]. The tumor milieu is dynamic, prosperous in diverse cell lineages, and characterized by hypoxia, low pH, and stromal cell complexity [48]. CD8^+^ T cells and natural killer (NK) cells perform anticancer activity through perforin-mediated cytotoxicity. Still, their activity is repressed by regulatory T cells (Tregs), IL-17-secreting γδ T cells, and immune-repressive M2 macrophages, neutrophils, and MDSCs, while B cells, dendritic cells, and mast cells exhibit dual pro- or anti-tumor activity depending on their secretory programs [49]. Chemotherapy resistance is associated with amplified LAG3^+^ exhausted T cells and decreased macrophage repolarization. Mesenchymal-like subtypes with elevated expression of TIM-3, VISTA, and TGF-β [50]. Myelopoietic-based populations are core modulators of immunosuppression. TAMs, like CD206^+^ M2-like subsets, promote immune tolerance through PD-L1 and IL-10, while MDSCs repress T and NK cell action via S100A8/A9 and PD-L1-facilitated cascade and ultimately augment resistance to therapy [51]. Neutrophils, along with tumor-associated neutrophils, ease angiogenesis, invasion, and immune suppression mediated by NET formation and PD-L1 expression, with CXCL5/CXCR2 signaling associated with reduced susceptibility to anti-PD-1 therapy [52]. Prolonged *H. pylori* infection further worsens inflammation and tumorigenesis via CCL2-mediated macrophage [53] employment and TNF-α/WNT and EGFR cascade.

**Fig. 2:**
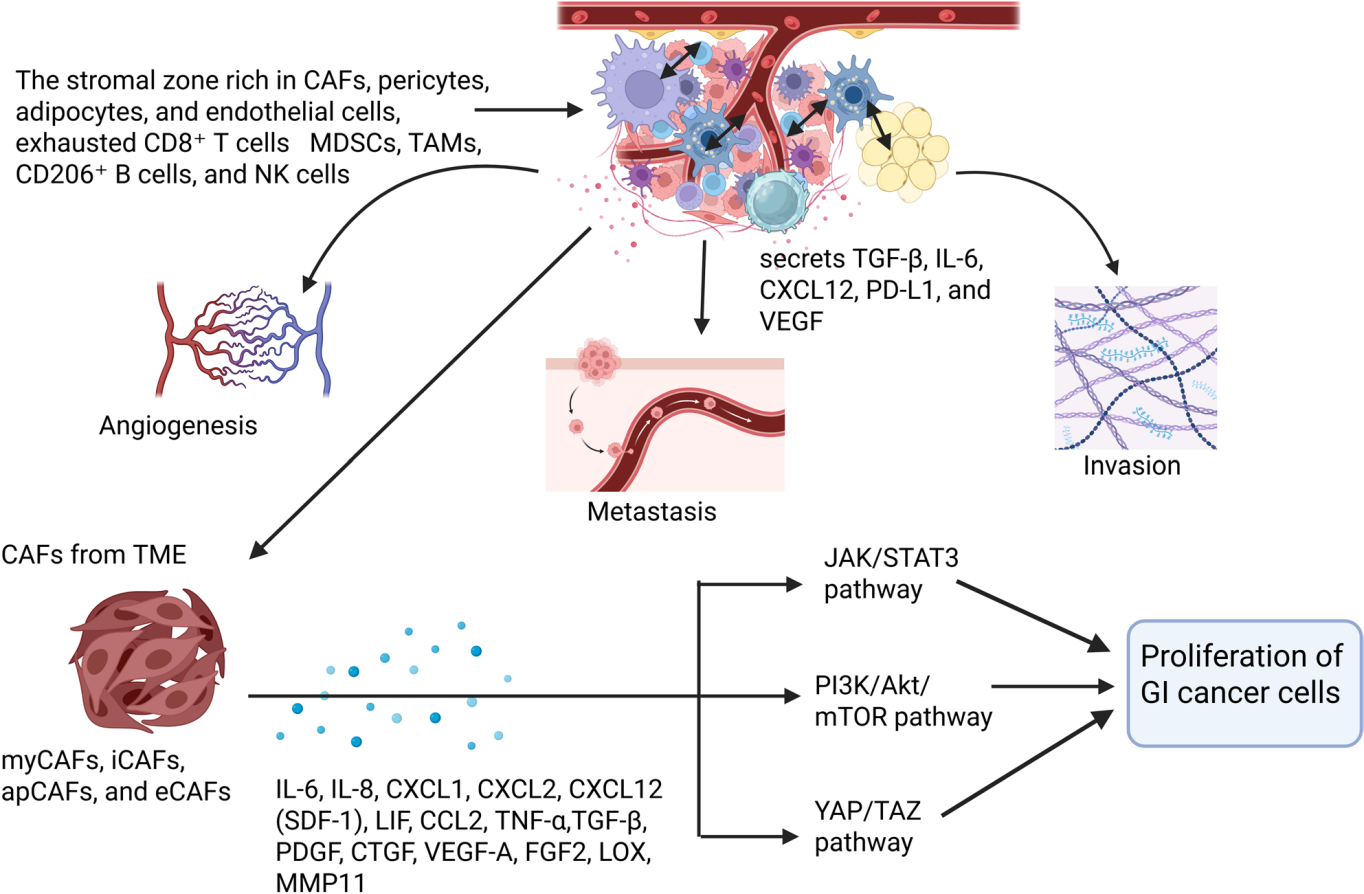
The complex interaction between tumor microenvironment (TME) components —pericytes, cancer associated fibroblasts (CAFs), tumor associated macrophages (TAMs), T cells, and B cells— establishes a complex paracrine signaling network via secreted factors TGF-β, IL-6, CXCL12, PD-L1, and VEGF, which promote angiogenesis, ECM remodeling, invasion, and metastasis. The CAF subtypes present in the TME, as shown in the figure, including myCAFs, iCAFs, and apCAFs, release cytokines and growth factor mediators IL-6, IL-8, CXCL1, CXCL2, CCL2, TNF-α, and β, PDGF, etc., remodel the ECM, increase tumor stroma interactions, and maintain metastasis. The secreted growth factors trigger JAK/STAT3, PI3K/Akt/mTOR, and YAP/TAZ signaling, maintaining proliferative pathways

The ECM, along with a structural scaffold, is a dynamic signal hub that controls gene expression, cell proliferation, and invasion via specialized binding sites that sense its composition and stiffness [54]. Fibroblasts and CAFs maintain ECM homeostasis by producing collagens, fibronectin, laminins, and proteoglycans, whilst secreting matrix remodellers such as MMPs (Fig. 2) [49], LOX, cathepsins, and heparanases, which are offset by TIMPs [55]. In cancer, CAF-facilitated ECM stiffness mediated by collagen I crosslinked through LOX [56] and deposition of collagens VI, XI, XXII, and XXIV triggers mechano transduction pathways like YAP/TAZ, which strengthen cytoskeletal contractility through ANLN [57], DIAPH3, and drive a positive feedback loop of matrix stiffness, proliferation, and resistance to therapy [58]. CAFs also secrete DKK3, which synergizes with YAP/TAZ and the Wnt/β-catenin signaling pathway to increase tumor aggressiveness [59]. Remodeling of ECM architecture promotes angiogenesis and metastasis; fibronectin association and podoplanin facilitate actomyosin contractility, establishing “tracks” that guide tumor cells, while DDR2-dependent CAF–collagen interactions enable metastasis [60]. CAF-induced secretion of cytokines IL-6, CXCL12, integrates ECM remodulation with immune tolerance and stroma-tumor crosstalk (Fig. 2) [61]. The dual role of αSMA+ myCAFs or meflin-positive CAFs displays context-dependent balance between pro- and anti-tumor ECM dynamics [62]. CAFs reprogram cancer metabolism by delivering nutrients and exosomal cargos such as lncRNA SNHG3, to increase glycolysis, boosting PGE2, CXCL12/CXCR4, and NNMT to maintain proliferative signaling [63]. CAFs secrete VEGFA, FGF2, PDGFC, CXCL12, CTGF, and CHI3L1, promote angiogenesis, employ myeloid cells and endothelial precursors, and promote vascular mimicry through Notch2/Jagged1 signaling, a contributor to anti-VEGF resistance [64]. CAFs also remodel invasion and metastasis by aligning fibronectin fibers via α5β1 integrins and PDGFRα, facilitating EMT and invasion through CXCL12, TGF-β, NOTCH, and RHBDF2 pathways, and transforming into metastasis-associated fibroblasts (MAFs) to remodel niches [65]. Chemokines CXCL5 and CCL5, growth factors FGF2 and MFAP5, and interleukins IL-6, IL-32, and IL-33 promote CAFs and enhance metastatic dissemination by recruiting neutrophils, stabilizing the HIF1α/ZEB1 signaling axis, and triggering integrin/MAPK cascades [66]. CAFs also synthesize collagens, proteoglycans, and glycoproteins while releasing MMPs and LOXL2, which are responsible for generating stiffened, aligned matrices that facilitate invasion, immune tolerance via PD-L1 upregulation, and metastatic colonization [67]. The ECM, remodeled by CAFs via collagen deposition, crosslinking, proteolytic remodeling, cytokine, and chemokine secretion, and exosomal cargo transfer, acts as a dynamic signaling hub that sustains cancer progression in a context-dependent, pro- and anti-tumor manner.

### Autophagy in cancer

Ability of cells to adapt to the stressful conditions in TME, including metabolic stress, trigger adaptive cellular mechanisms determines whether the cell survives or undergoes programmed death [68]. A core regulation of autophagy is a preserved lysosomal degradation mechanism in which autophagosomes engulf damaged proteins, aggregates, cytoplasmic content, and organelles, followed by lysosomal degradation, salvaging nutrients and maintaining energy homeostasis [69]. Basal autophagy complemented with the ubiquitin–proteasome–based degradation and prevents toxic accumulation [70]. Autophagy clears pathogens and apoptotic cells and exposes processed peptides to antigen-presenting cells [71]. Autophagy defects lead to imbalanced homeostasis and contribute to cancer development [72]. Nutrient undernutrition inactivates TOR kinase, causing hypo-phosphorylation of Atg13, which then interacts with Ulk1/Atg1 and Atg17 to initiate phagophore assembly. Atg9, regulated by Atg1, imports lipids from various organelles, including the ER, Golgi, and mitochondria, contributing to membrane expansion [73]. Elongation is mediated by the Vps34/ Beclin1 complex, which produces PI3P to employ effector molecules WIPI1/2 and DFCP1, with regulation of Ambra1, UVRAG, Bif-1, and decreased Bcl-2, Bcl-xL, and Rubicon [74]. The Atg12–Atg5–Atg16L complex induces curvature, and the LC3; LC3 is cleaved by Atg4, lipidated to LC3-II, and introduced into the membrane to initiate elongation [75]. LC3-II mediates cargo selection, cooperates with p62/SQSTM1 adaptor proteins target polyubiquitinated aggregates and damaged organelles, mitophagy clears mitochondria [76]. Mature autophagosomes are transported along microtubules toward lysosomes, where Rab7, SNAREs, ESCRT, and class C Vps proteins coordinate vesicle fusion, facilitated by UVRAG, while Rubicon suppresses it [77]. Fusion with lysosomes results in the production of acidic hydrolases that degrade sequestered material and release amino acids and fatty acids under stress conditions. Prolonged stress can trigger autophagy, leading to type II programmed cell death (autophagic cell death), a caspase-independent cell death [78]. In cancer, autophagy has a dual role at early stages, acting as a tumor suppressor by preventing genomic instability, clearing damaged organelles, and averting the accumulation of oncogenic signals [79]. At advanced stages, cancer cells manipulate autophagy as a tumor-promoter and utilize it in nutrient deficiency, hypoxia, and stress-induced therapy, thus increasing aggressiveness and resistance to apoptosis.

Autophagy exhibits a context-dependent role in tumor initiation and progression, as basal autophagic activity is reduced in cancer cells, causing increased genomic instability and cancer transformation [80]. Genes like PTEN downregulate the PI3K/Akt/mTOR axis by transforming PIP3 to PIP2, and loss of PTEN leads to hyperactivation of the Akt signal, a suppressor of autophagy, while influencing protein synthesis and tumor growth [81]. A tumor suppressor gene, nuclear p53, activates autophagy via AMPK and TSC1/TSC2-mediated signal; on the contrary, cytoplasmic p53 inhibits autophagy, and mutations in p53 consequently disturb this equilibrium, supporting cancer progression [82]. p53’s downstream effector, damage-regulated autophagy modulator, is required for p53 to facilitate autophagy and apoptosis, and its loss causes cancer progression [82, 83]. DAPK, a calmodulin-regulated serine/threonine kinase, represses tumor growth and metastasis by promoting p53-associated apoptosis and autophagy; loss of DAPK reduces autophagy and enhances invasiveness [84]. LKB1/AMPK, an energy-sensing signal, activates TSC2 and inhibits mTOR under metabolic stress, induces autophagy, and conserves energy through cell cycle arrest [85].

Oncogenes suppress autophagy to promote tumorigenesis, as PI3K-Akt-mTOR is overactivated in cancers, and it inhibits autophagy [86]. Bcl-2 family proteins bind to the BH3 domain of Beclin1, shielding it from Vps34 and blocking autophagosome formation; overexpression of Bcl-2 suppresses autophagy, while its suppression restores autophagic flux [87]. FLIP and mTOR also inhibit autophagy, thereby increasing cell survival and therapy resistance. Beclin1 itself acts as a core, as its interactions with cofactors such as UVRAG and Bif-1 activate autophagy through Vps34, whereas Rubicon downregulates Beclin1 function [88]. Mutations or loss of Beclin1, UVRAG, or Bif-1 reduce autophagic activity and encourage tumor progression [89]. Dysfunction of ATG5/7/4 C and ATG16L1 weakens mitochondrial function, leading to elevated ROS, DNA impairment, and an inflammatory signal that enables malignancy [90]. ULK3/Atg3-regulated autophagy stimulates senescence and inhibits proliferation, while inhibition of autophagy delays senescence and enhances oncogenic potential [91].

Oxygen deficiency and metabolic strain in tumors activate autophagy via the HIF-1α and AMPK signaling pathways, which limit necrosis, downregulate inflammation, reduce macrophage infiltration, remove p62, and avert ROS accumulation, DNA impairment, and genomic variability [92]. PERK kinase signaling facilitates autophagy in ECM-degraded cells, maintaining redox balance and promoting anoikis resistance during dissemination [93]. Autophagy facilitates metabolic rewiring by providing substrates for glycolysis, glutaminolysis, and the TCA cycle, with mitophagy preserving mitochondrial function [94]. In RAS-mediated tumors, elevated basal autophagy maintains mitochondrial function and supports growth and persistence, with HIF-1α and AMPK/mTOR signaling under nutrient- and stress-driven tumor milieu [95]. AEG-1 oncogenes activate AMPK-mTOR-associated autophagy, and ATG5/7 autophagy gene knockdown sensitizes cancer cells to cellular stress and therapy. Autophagy supports metastasis by enabling survival under stress, evasion of TRAIL-induced apoptosis, and sustained metabolic plasticity [96, 97]. In tumor dormancy, autophagy is regulated by β1-integrin signaling, which induces autophagy in poor ECM connections, permitting disseminated tumor cells to survive in a quiescent state [98]. In bone metastasis, Src-mediated TRAIL resistance and autophagy facilitate the long-term survival of dormant disseminated tumor cells, whereas in ovarian cancer, ARHI-induced autophagy mediates dormancy rather than cytotoxicity, depending on microenvironmental cues [99].

Autophagy also modulates therapy outcome through numerous pathways, as autophagy induced by chemotherapeutic stress involves Beclin1, LC3 lipidation, ATG protein activation, and JNK/BCL2 signal maintains survival and results in resistance [100]. Inhibition of these pathways by 3-MA, chloroquine, hydroxychloroquine, or genetic silencing of ATG proteins reestablishes apoptosis sensitivity and enhances the efficacy of chemotherapy to 5-FU, TRAIL, and targeted therapies [101]. Autophagy mediates drug resistance in cancer; for example, chemotherapeutic agents such as 5-FU and cisplatin induce autophagy via Beclin 1 upregulation, LC3 lipidation, ATG7 expression, and JNK/Bcl-2 signaling, which diminishes apoptosis and therapy efficacy [102]. It also modulates immune tolerance by activating STAT3 under hypoxia, thereby protecting cancer cells from T-cell cytotoxicity. In cancer stem cells, autophagy tolerates self-renewal, stemness, and existence under metabolic or hypoxic stress. In glioma cells with self-renewal capacity, autophagy promotes cellular differentiation and survival, whereas in breast CSCs it preserves enhanced motility and invasiveness [103]. In CRC, autophagy preserves pluripotency and proliferation via ATG5/7, while blockade decreases stemness markers Oct4, SOX2, and Nanog, persuading senescence and sensitizing CSCs to treatment therapy [104]. Thus, autophagy activation is context-dependent: in the initial stage, it acts as a tumor suppressor, maintaining genomic stability, but could be hijacked by advanced cancers and CSCs through proliferative pathways to support metabolic alterations and survival under stress.

### Caloric restriction (CR) is an autophagy-controlling intervention

CR and fasting-mimicking regimens have recently been recognized as systemic approaches to induce autophagy, which could also affect tumor biology independently of cancer cell-autonomous mechanisms [105]. CR, through the reduction of circulating glucose, insulin, and IGF-1, and the activation of AMPK with suppression of mTOR signaling, could increase autophagic flux in both tumor and stromal cells [106]. In GI malignancies, this systemic metabolic change could affect CAF activation, the ECM, and the metabolic interactions between CAFs and malignant cells. For example, nutrient deprivation could affect lactate and lipid transport, reduce the secretion of pro-tumorigenic cytokines, and reorganize immune cell infiltration in the TME [107]. However, because autophagy has been shown to have context-dependent pro-survival and tumor-suppressive roles, the effect of CR on CAF-malignant cell interactions would be complex and likely dependent on the specific malignancy type and stage. Thus, well-designed preclinical and clinical studies are required to determine whether metabolic therapy can be safely used to modulate autophagy in stromal cells and improve therapeutic outcomes in GI malignancies.

### Autophagy in CAFs

Autophagy is normalized by a coherent signal cascade that senses stress due to nutrient deprivation and energy imbalance and translates this into coordinated membrane biogenesis, cargo sequestration, and lysosomal degradation [108]. Upstream, mTORC1 incorporates with growth factor and nutrient signals to phosphorylate and inhibit the ULK1-ATG13-FIP200 initiation complex under nutrient-dense conditions, whereas AMPK, which is activated by low ATP and HIF-1α triggered by hypoxia, inhibits mTORC1 and/or directly activates ULK1 to induce autophagy [109]. The triggered ULK1 complex recruits the VPS34/Beclin1/ATG14/VPS15 class III PI3K complex to produce PI3P on membranes, engaging WIPI-ATG18 proteins and DFCP1 to nucleate phagophores [100]; Beclin1 activity is positively modulated by UVRAG and Bif-1 and negatively modulated by Bcl-2/Bcl-xL and Rubicon [110]. Phagophore elongation depends on membrane transport by ATG9 and ATG12–ATG5–ATG16L1, a scaffold/E3-like activity, and LC3-ATG8 lipidation. LC3-I mediates LC3-II via ATG7/ATG3, which mediate membrane closure and create LC3-decorated autophagosomes that recruit p62/SQSTM1, NDP52, NBR1, and cargo via receptors carrying LIR motifs [111]. Autophagosome trafficking and fusion with endolysosomal compartments need Rab-GTPases, Rab7, SNAREs, ESCRT system, and also microtubule and dynein transport; lysosomal based hydrolysis releases amino acids and fatty acids to restore metabolic homeostasis [112]. In cancer, the autophagy regulation rewired the core oncogenic pathway PI3K/Akt/mTOR signal downregulate ULK1 and autophagy start, while RAS-driven tumors elevate basal autophagy through elevated mitophagy to withstand bioenergetics and redox balance [113]; ER stress with PERK signal promotes autophagy and radical stress responses to allow anoikis resistance; JNK/BCL2 and STAT3 pathways effect the equilibrium between protective autophagy and apoptosis or immune tolerance [114]. BECN1, ATG5, ATG7 mutation or loss of function dysfunctions mitochondrial activity, upsurges ROS and genomic variability, stimulates tumor initiation; HIF-1α-AMPK activation; autophagy mediated survival in hypoxic, nutrient-poor niches, dormancy, metastasis, and therapy resistance [115].

**Fig. 3:**
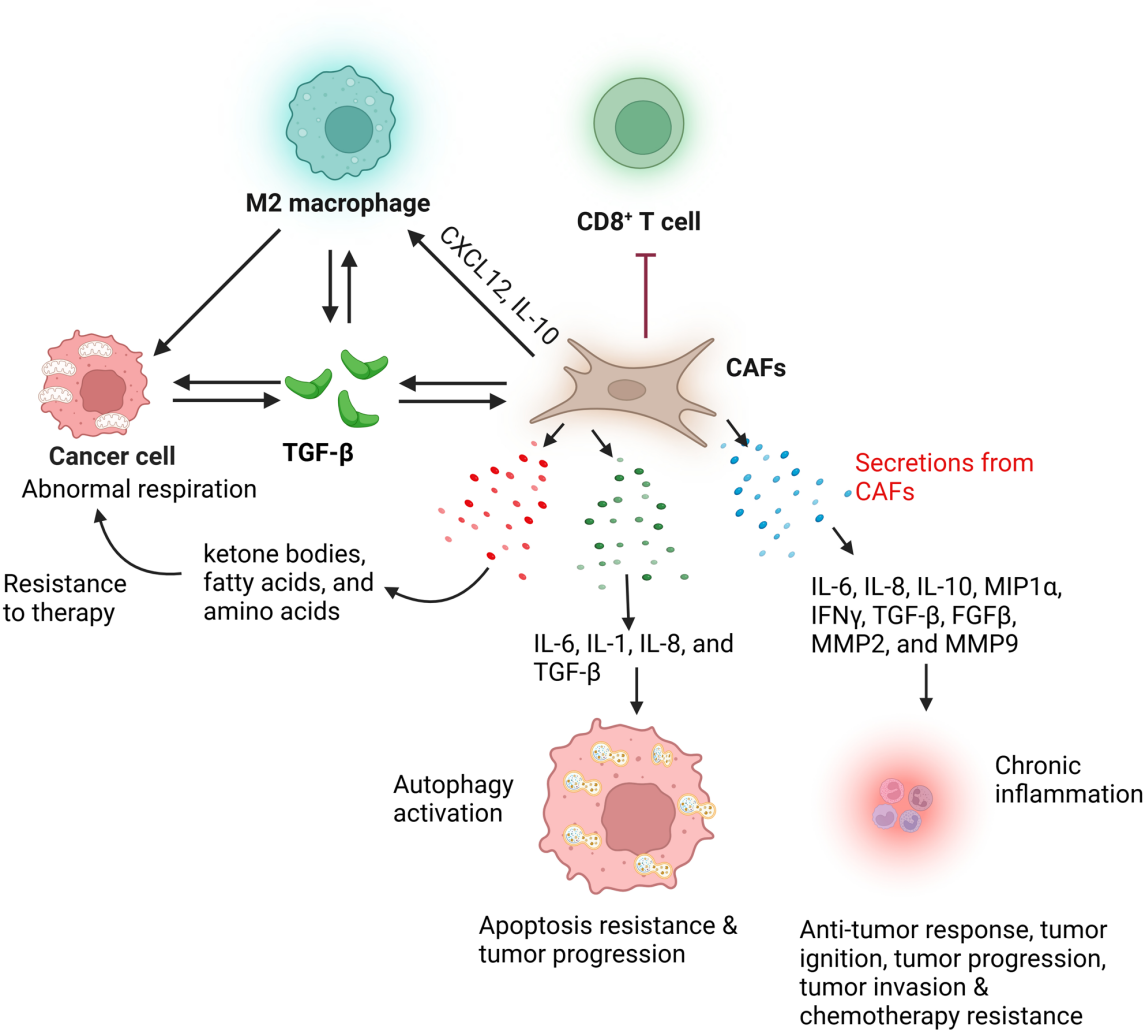
Interaction of cancer associated fibroblasts (CAFs) and other tumor microenvironmental (TME) components. M2 Macrophages, cancer cells, and CAFs secrete tissue growth factor beta (TGF-β), which is essential for CAFs activation and tumor growth progression. CAFs inhibit CD8^+^ T cells and stimulate M2 polarization via the secretion of CXCL12 and interleukin-10 (IL-10). CAFs secrete various growth factors and cytokines that are involved in mitochondrial dysregulation in cancer cells, autophagy activation, and the induction of chronic inflammation. These three types of signaling induce cancer cells to become resistant to chemotherapy, to resist apoptosis, and to undergo autophagy, thereby promoting tumor progression

Inside the tumor milieu, CAFs are activated by paracrine signals through TGF-β, IL-1, PDGF, and IL-6 and by ECM to adopt distinct phenotypes: myCAFs, through TGF-β/SMAD3 signal, induce α-SMA⁺ high contractility and elevate autophagy genes BECN1, ATG7 through collaboration with chromatin modifiers MYST1 downregulation; iCAFs ascend when IL-1/IL-6/LIF activate IL-1R–JAK/STAT and NF-κB, stimulating an inflammatory secretome and autophagy via ATG transcription, ROS/AMPK–mTORC1 regulation and Notch-dependent juxtacrine signaling that strengthens fibroblast to CAF transformation [116]. CAF diversity involves quasi EMT, epigenetic rewiring and metabolic remodel; CAFs supply metabolites, cytokines and pro-survival signals including IL-6/STAT3 that nourish tumor cell autophagy and support tumor cell metabolic flexibility [117]. On the contrary, CAF autophagy can release metabolites and immunomodulatory factors that reinforce immune tolerance through apCAFs, inducing Tregs and tumor growth (Fig. 3). Thus, the array of interconnected signal wires ULK1/VPS34/ATG system in tumor cells and TGF-β/IL-6/PDGF-facilitated CAF activation in the stroma, which reciprocates a metabolic and immunoregulatory network that controls whether autophagy acts as a tumor suppressor or tumor promoter [118]. A thickened ECM trigger integrin stimulates FAK/Src and remodel cytoskeleton, which triggers MRTF-SRF and YAP/TAZ–TEAD transcription. The nuclear YAP/TEAD facilitates matrix fabrication, metabolic rewiring and ultimately promotes autophagy by upregulating cellular demand and by controlling AMPK/mTOR signal. DKK3-HSF1 stress sensors and DNA damage induced secretion of activin-A auxiliate stability YAP/TAZ and tolerate the autophagy permissive transcriptional state [119, 120]. Canonical autophagy incorporates upstream signals to activate the ULK1/ATG13/FIP200 complex, recruit VPS34–Beclin1–ATG14 for PI3P fabrication, mobilize membranes via ATG9, and facilitate phagophore expansion through ATG12–ATG5–ATG16L1 and LC3-II lipidation (ATG7/ATG3) [121]. CAF autophagy withstands a secretory and metabolic program, releasing cytokines, HMGB1, ECM-remodel enzymes, and lactate, alanine, fatty acids to support tumor survival and progression, and CAFs strengthen under hypoxia and nutrient stress [122]. This autophagy facilitates secretome and metabolic coupling, enhancing tumor growth, immune modulation through apCAF-induced Tregs, and matrix remodeling, developing a feed-forward loop that maintains CAF activation and continuous autophagy.

The CAFs are specified stromal cells in TME and are different from normal fibroblasts. The identification can be done based on the markers, which are highly expressed, including FAP-α indicates activated fibroblasts [123]; α-SMA are contractile, myCAFs; PDGFRα/β involved in CAF proliferative signal; Vimentin a cytoskeletal marker of mesenchymal cells; MYL9, MYLK, MMP2, COL1A2 are ECM remodeler and contractile; transgelin; periostin; podoplanin; FSP1 perform CAF subpopulations. The negative markers include EPCAM used to eliminate epithelial cells from CAFs [124]. Tumor cells secrete factors that stimulate autophagy in CAFs, and reprogram to maintain tumor progression [125]. In PDAC, VDR stimulates LC3A/B signal, which promotes CAF autophagy, increasing tumor progression [126]. Hydrogen peroxide stimulates NF-κB and ROS cascade that fuels mitochondrial biogenesis and delivers energy substrates, and miRNA modulation increases aggressiveness of cancer [127]. Moreover, HIF1α mediated through NF-κB, enhances autophagy under hypoxia. TGF-β activation through ROS signaling drives autophagy, increasing metastasis in breast cancer.

In PDAC, the IL-6 activates STAT3, which induces CAF autophagy [128]. In oral squamous cell carcinoma, the TGF-β, activated through the SMAD signal, activates autophagy [129]. Along with Lnc-CAF downregulation and IL33 upregulation, which favor tumor progression through autophagy [129]. The implicated signal transduction pathways include CAFs integrating through TGF-β, IL-1, IL-6, PDGF, and ROS [130]. STAT3 promotes transcription of autophagy-related genes [131]. AMPK identifies metabolic stress and activates autophagy. NF-κB regulates inflammatory and stress-induced autophagy [132]. SMAD mediated TGF-β-mediated transcriptional activation of autophagy genes like BECN1 and ATG7 [133].

### Autophagy-associated CAF in GI malignancies

In this, we discuss the numerous studies performed on autophagy in GI cancers. A recent study by Zhang et al. [134] found that CAF autophagy supports tumor growth in PDAC and plays a role in immune regulation (Fig. 3). Blocking CAF autophagy reduces tumor-promoting signals, including IL-6 and desmoplasia, but it also increases PD-L1 levels, which can weaken anti-tumor immune response. USP14 suppresses PD-L1 via deubiquitination, and loss of CAF autophagy reduces USP14 [116, 135, 136]. Using chloroquine-loaded MSC-liposomes to target CAFs enhanced the response to immune-chemotherapy, suggesting CAF autophagy is both a regulator of the TME and a potential therapeutic target [137]. CAFs help PDAC survive under glutamine deficiency by secreting nucleosides through NUFIP1-dependent autophagy. These nucleosides boost glucose utilization and tumor growth via a MYC-dependent mechanism. Blocking nucleoside release by targeting NUFIP1 reduced tumor burden, revealing a key CAF-driven metabolic support system in PDAC [138]. Another study reported that autophagy in CAFs drives their activation by promoting proline biosynthesis and collagen production through mitophagy-dependent regulation of NADK2. Blocking mitophagy via PRKN inhibition in CAFs reduced tumor growth in PDAC [139]. The study revealed that high CAF density in PDAC is associated with stronger inflammatory signaling and enhanced EMT in tumor epithelial cells. Using transfer learning, transcriptional data from patient-derived organoid CAF co-cultures confirmed that CAFs directly induce these epithelial states. Functional validation determined that CAF–tumor cross-talk mediated through ITGB1 and VEGFA interactions with neuropilin-1, drives EMT and inflammation. Thus, highlighting the utility of integrating human scRNA-seq data with co-culture models to reveal clinically relevant CAF–tumor interactions that sustain PDAC progression (Fig. 3) [140]. CAFs in PDAC stroma drive tumor progression and therapy resistance through palladin and CD146 markers, exosomal and cytokine signaling, including GM-CSF, IL-6, and signaling through pathways like JAK/STAT, mTOR, SHH, and NF-κB. CAF autophagy and metabolism further enhance tumor survival in nutrient-poor environments (Fig. 3). Thus, targeting CAF functions or stromal pathways is emerging as a promising therapeutic strategy in PDAC [141]. A distinct CAF subpopulation in HCC using single-cell sequencing found that YAP1 + CAFs are the drivers of matrix stiffness. These cells upregulate stiffness-related genes *COL1A1*,* COL3A1*, and *LOX;* activate autophagy and GTPase pathways, and promote tumor progression. High expression of these genes correlates with poor prognosis in HCC patients [142]. A study shows that miR-31 is upregulated in CRC CAFs, and its overexpression inhibits autophagy-related genes Beclin-1, ATG, DRAM, and LC3 in CAFs, thereby affecting CRC progression and apoptosis regulation, while also increasing the radiosensitivity of CRC [143]. lncRNA FAL1 is overexpressed in CRC and associated with poor survival. CAF-derived exosomes deliver lnc-FAL1 to cancer cells, where it suppresses oxaliplatin-induced autophagy by promoting TRIM3-mediated Beclin1 degradation and enhances chemoresistance to oxaliplatin in CRC [144]. CAFs in the TME enhance CRC metabolism. This effect mediates through CAF autophagy and oxidative stress pathways, which are activated by signals from adjacent tumor cells. Targeting these fibroblast-mediated pathways could offer a novel strategy for CRC therapy [145]. A study revealed that loss of HIPK2 in cancer cells promotes the trans-differentiation of normal fibroblasts into CAF-like cells via conditioned media. This CAF conversion is characterized by α-SMA and collagen I expression and involves autophagy-mediated degradation of caveolin-1 [146]. A blue LED light (465 nm) suppresses CRC growth by enhancing autophagy in CRC and reducing CAF activation. LED irradiation decreases CAF markers α‑SMA, IL‑6, TGF‑β, and PD‑L1 expression [112]. CAFs also promote peritoneal metastasis in gastric cancer by releasing cancer-promoting cytokines and by resisting chemotherapy. The oncolytic adenovirus OBP-702 delivers wild-type p53, stimulates apoptosis and autophagy in CAFs, diminishes cytokine secretion, and neutralizes gastric cancer cells. Combined with paclitaxel, OBP-702 synergistically hinders tumor proliferation and decreases CAFs [147]. GALNT6 mediates lenvatinib resistance in HCC by promoting O‑GalNAc glycosylation, improving autophagy, and activating CAFs via the PDGFA/PDGFRB/SPP1 axis. GALNT6 knockdown reduces glycosylation and autophagy, hinders CAF activation, and restores lenvatinib sensitivity; thus, GALNT6 is a potential target to improve HCC therapy [148]. A study revealed that CAFs promote tumor relapses after radiotherapy by secreting IGF1/2, CXCL12, and β-hydroxybutyrate, which induce autophagy in irradiated cancer cells. This phenomenon occurs via elevating ROS levels, activating PP2A, inhibiting mTOR, and enhancing autophagy [149]. Thus, CAF-facilitated autophagy is important for tumor progression, therapy resistance, and TME modulation across GI cancers. Table 1 summarizes the clinical findings.

**Table 1:** Summary of autophagy-associated CAFs in GI malignancies

GI cancers	CAF autophagy implication	Mechanism	Effect on the tumor and therapy	Reference
PDAC	Supports tumor progression and immune regulation	IL-6, USP14, PD-L1; targeted with chloroquine MSC-liposomes	Reduces tumor-promoting signals, enhances immunochemotherapy response	[137]
PDAC	Improves survival under glutamine deprivation	Nucleosides secreted via NUFIP1-dependent autophagy; MYC pathway	Promotes glucose utilization and tumor growth; targeting NUFIP1 reduces tumor burden	[138]
PDAC	Drives CAF activation	Mitophagy mediated NADK2 regulation	Enhances proline biosynthesis, collagen production; PRKN reduction decreases tumor growth.	[139]
PDAC	Induces EMT and inflammation	ITGB1 & VEGFA neuropilin-1 signaling	Enhances tumor progression	[140]
PDAC	Enhances tumor progression and therapy resistance	Palladin, CD146, GM-CSF, IL-6; JAK/STAT, mTOR, SHH, NF-κB	Supports survival in nutrient-deprived environments; targeting CAF functions is therapeutic	[141]
HCC	Promotes matrix stiffness	YAP1plus CAF*s*,* COL1A1*,* COL3A1*,* LOX*, autophagy, GTPase pathways	Drives tumor progression, stiffness-gene expression	[142]
CRC	Modulates autophagy	miR-31 inhibits *Beclin-1*,* ATG*,* DRAM*,* LC3*	Affects tumor progression and apoptosis, and increases radiosensitivity	[143]
CRC	Induces chemoresistance	CAF derived exosomes secrete lnc-FAL1; TRIM3-mediated Beclin1 degradation	Enhances oxaliplatin resistance	[144]
CRC	Enhances metabolism	CAF autophagy and oxidative stress pathways	Promotes tumor growth under stress	[145]
CRC	CAF trans-differentiation	Loss of HIPK2 in tumor cells; autophagy-mediated caveolin-1 degradation	Converts normal fibroblasts to CAF-like cells	[146]
CRC	Reduces CAF activation	Blue LED irradiation	Suppresses tumor growth, decreases CAF markers, and limits PD-L1	[150]
Gastric Cancer	Promotes peritoneal metastasis	Cytokine secretion, chemo-resistance	Induction of apoptosis/autophagy in CAFs	[147]
HCC	Mediates drug resistance	GALNT6 mediated O-GalNAc glycosylation; PDGFA/PDGFRB/SPP1; autophagy	Activates CAFs, promotes lenvatinib resistance; knockdown restores sensitivity	[148]
Various GI Cancers	Promotes relapse after radiotherapy	IGF1/2, CXCL12, β-hydroxybutyrate increase ROS activates PP2A, mTOR inhibition regulates autophagy	CAFs aid irradiated tumor recovery; targeting autophagy reduces relapse	[149]

### Microbiota in the regulation of autophagy and CAF behavior in GI cancers

The GI microbiota is an important factor in determining tumor biology through the regulation of inflammation, metabolism, and host signaling pathways that interact with autophagy [151, 152]. Microbiota-derived metabolites, including short-chain fatty acids (SCFAs), secondary bile acids, and lipopolysaccharides, can directly affect autophagy-related signaling pathways mediated by AMPK, mTOR, and NF-κB in epithelial and stromal cells [152, 153]. In CRC and GC, microbiota dysbiosis-induced chronic inflammation leads to the production of cytokines (IL-6 and TGF-β), which not only trigger autophagy in tumor cells but also promote the activation and differentiation of fibroblasts into CAFs (Fig. 3) [152]. Moreover, specific microbial signatures have been linked to changes in the metabolic status of the TME, which may sustain autophagy-dependent survival pathways during nutrient deprivation.

In addition to tumor cells, the microbiota can modulate CAF behavior by regulating stromal metabolism, ECM remodeling, and immune interactions [154]. Microbial components can induce oxidative stress and autophagy in fibroblasts, thereby facilitating their conversion to a tumor-supportive phenotype, characterized by increased production of growth factors, cytokines, and ECM components [145, 155]. Conversely, autophagy in CAFs can contribute to tumor growth by promoting metabolic cooperation, such as lactate and lipid transfer [156]. Notably, microbiota-mediated modulation of autophagy can also influence treatment outcomes, as changes in gut microbiota composition have been associated with the efficacy of chemotherapy and immune checkpoint blockade. Taken together, these observations indicate that microbiota-autophagy-CAFs interactions are another aspect of context-specificity in GI cancers and may provide new opportunities for therapeutic intervention.

### Metabolic coupling between malignant cells and CAFs (Warburg and reverse Warburg effects) in GI cancers

Metabolic coupling between malignant cells and CAFs is now being recognized as a major hallmark of autophagy-dependent tumor-stroma interactions in GI malignancies [122, 157]. This association is significantly affected by the Warburg and reverse Warburg effects. While the Warburg effect is commonly observed in cancer cells, which prefer aerobic glycolysis for energy and biosynthetic precursor production, the reverse Warburg effect is observed in CAFs, which exhibit a glycolytic phenotype and secrete lactate, pyruvate, and ketone bodies [158, 159]. Autophagy in CAFs supports this metabolic shift by degrading cellular components to generate metabolites, which are then transported to the oxidative cancer cells via monocarboxylate transporters (e.g., MCT4) [160]. This reciprocal metabolic interaction not only supports tumor growth under nutrient and oxygen deprivation but also maintains therapy resistance and stromal activation. Therefore, autophagy plays a pivotal role in regulating this metabolic interaction within the TME (Fig. 3).

Autophagy in CAFs enhances the degradation of cellular materials, producing metabolites that can be secreted to support neighboring cancer cells (Fig. 3) [122]. The major player in this metabolic cooperation is the lactate transport system mediated by monocarboxylate transporters, especially MCT4 in glycolytic CAFs [156, 161]. Lactate secreted from CAFs can be readily taken up by oxidative cancer cells and used in the tricarboxylic acid cycle, thus supporting ATP production and biosynthesis [162]. Likewise, ketone bodies produced through autophagy-mediated metabolic reprogramming may act as alternative energy sources, thus supporting tumor development even in hypoxic or nutrient-limited environments (Fig. 3) [163].

Besides carbohydrate metabolites, lipid exchange is another important dimension of the metabolic partnership between tumors and stroma. Autophagy may promote lipolysis and lipid mobilization in CAFs, making it easier for fatty acids to be transported to cancer cells (Fig. 3) [164]. Lipid uptake mediated by CD36 in cancer cells supports the efficient uptake of fatty acids, leading to β-oxidation, membrane biogenesis, and protection against metabolic stress [165, 166]. This metabolic adaptability, supported by lipid metabolism, is especially important in highly desmoplastic tumors such as PDAC and HCC, in which nutrient supply is limited [167, 168]. Notably, these metabolite transfers are not only supportive but also play a role in shaping the response to therapies, as metabolic plasticity can confer resistance to chemotherapy and autophagy-targeting therapies. Taken together, lactate and ketone transport, as well as CD36-mediated lipid uptake, demonstrate how autophagy coordinates metabolic and stromal signals to support tumor growth across various GI cancers.

### Hydroxychloroquine and chloroquine in GI malignancies and their translational relevance

From a translational point of view, the pharmacologic inhibition of autophagy has been an area of active investigation in GI malignancies with the use of lysosomal inhibitors such as hydroxychloroquine (HCQ) and chloroquine (CQ) [169]. These drugs, originally used in the treatment of malaria and autoimmune diseases, act by inhibiting autophagic flux due to the inhibition of lysosomal acidification and autophagosome breakdown [169]. In GI malignancies, HCQ and CQ have been investigated in early-phase clinical trials, mostly in combination with chemotherapy, targeted therapy, or radiation [170]. The underlying hypothesis is to inhibit the tumor cell recycling pathways that contribute to tumor survival under conditions of metabolic and therapeutic stress [171]. In PDAC, in which KRAS-driven tumors have been shown to have an increased reliance on autophagy, autophagy inhibitors have demonstrated biologic activity in combination regimens, although clinical responses have been inconsistent [126, 170, 172].

However, although preclinical results have been encouraging, translation to the clinic has revealed several key issues. The role of HCQ or CQ treatment appears to be context-dependent and may be influenced by tumor genotype, stromal content, and treatment timing [172]. Furthermore, most trials have focused primarily on the autophagic properties of tumor cells, with little attention to stromal contributions, such as CAFs. However, given the growing evidence that autophagy in CAFs contributes to metabolic and immunological coupling and to the development of therapy resistance, future clinical trials may need to address both the tumor and stromal components.

### Adenosine: a major hypoxia associated metabolite that affects the TME and CAF function in GI cancers

In the context of GI malignancies, adenosine has been identified as a major hypoxia-related metabolite that significantly affects the TME and CAF function [173]. In hypoxic environments, the ectonucleotidases CD39 and CD73 catalyze the conversion of extracellular ATP to adenosine, resulting in its accumulation within the TME [174]. High levels of adenosine contribute to immunosuppression by suppressing effector T cells and NK cells, while also promoting CAF activation, ECM remodeling, and metabolic reprogramming [37]. Moreover, recent findings indicate that adenosine signaling interacts with autophagy, influencing the regulation of both CAFs and tumor cells under stress conditions [175]. By integrating adenosine mechanisms with autophagy regulation, this model emphasizes the roles of hypoxia and metabolite signaling in coordinating stromal and tumor cell responses in GI cancers.

### Therapeutic effects of targeting autophagy in CAFs of GI cancers

The role of autophagy in CAFs is critical for tumor sustenance, metabolic coupling, immune evasion, and therapeutic resistance in GI cancers [176]. Thus, targeting autophagy in CAFs has been explored as a promising approach to enhance the efficacy of conventional, immunotherapeutic, and combination therapies (Table 2). Lysosomal inhibitors, such as HCQ and CQ, have been evaluated in preclinical and early-phase clinical trials as pharmacologic agents to inhibit autophagy in combination with chemotherapy and targeted therapies [177]. In addition to lysosomal inhibitors, metabolic therapies such as caloric restriction, fasting-mimicking diets, and lactate/lipid shuttle inhibitors may affect autophagy-dependent stromal support and tumor-CAF metabolic coupling [37]. These approaches aim to selectively modulate autophagy in stromal cells to reduce pro-tumorigenic signals and improve therapy response. Taken together, these studies indicate that the modulation of autophagy in CAFs may have an impact on the TME, as well as the potential to improve the efficacy of traditional and immunotherapeutic approaches. Further research is required to determine the most effective combination of drugs, as well as the effects in various GI malignancies.

**Table 2:** Preclinical and clinical studies of autophagy-targeting strategies in GI cancers, including their mechanisms, cancer types, and therapeutic settings

Drug	Target/Mechanism	Type of GI cancers	Study type	Findings	References
Hydroxychloroquine	Lysosomal autophagy inhibition	PDAC	Phase I/II clinical trials	Safe in combination with chemo; evidence of altered PDAC metabolism; modest clinical activity.	[126, 178, 179]
Chloroquine	Lysosomal autophagy inhibition	CRC	Preclinical & clinical	Improves chemosensitivity; reduces autophagic flux in stroma; improves antitumor effects.	[180, 181]
Caloric restriction / Fasting	Systemic autophagy induction and metabolic stress	PDAC, CRC	Preclinical	Reduces CAF metabolic support; raises chemotherapy sensitivity; influences immune microenvironment.	[182, 183]
MCT4 inhibition	Blocks lactate export (reverse Warburg)	PDAC, CRC	Preclinical	Interrupts CAF-to-tumor lactate shuttling; decreases tumor growth; sensitizes to chemotherapy.	[184, 185]
CD36 inhibition	Blocks fatty acid uptake in tumor cells	HCC, PDAC	Preclinical	Dysregulates lipid metabolic coupling; decreases metastatic potential; increases the response to chemotherapy.	[186, 187]
Hydroxychloroquine+ chemo/targeted agents	Dual autophagy targeting	PDAC, CRC	Clinical / Early Phase	Metabolic changes consistent with autophagy inhibition; ability for enhanced chemo response; needs biomarkers.	[179, 188]

### Autophagy in CAFs affects monotherapy, immunotherapy, and combination therapy

The role of autophagy in CAFs has been identified as a key factor in the regulation of treatment response in GI cancers [189]. In the context of mono-therapies, such as chemotherapy or radiation therapy, autophagy in CAFs could increase tumor cell survival through the provision of metabolic substrates, the promotion of detoxification of reactive oxygen species, and the reinforcement of the ECM barrier [134, 190]. In the context of combination therapies, autophagy in CAFs could regulate sensitivity to targeted therapies or combination therapies through the regulation of nutrient and lipid flux, the support of metabolic plasticity, and the maintenance of a tumor-supportive ME [134, 190]. In the context of immunotherapy, autophagy in CAFs could promote immune evasion through the accumulation of adenosine, cytokine-mediated T-cell suppression, and metabolic competition with effector immune cells [191, 192]. Hypothetically, therapies that selectively target autophagy in CAFs could concomitantly decrease metabolic support for malignant cells, normalize the tumor stroma, and increase immune infiltration, thereby improving treatment response in mono-, combination-, and immunotherapies. Future studies will be necessary to determine biomarkers that predict which patients and tumor types will benefit most from the modulation of autophagy in CAFs.

## Conclusion

GI cancers, such as esophageal cancer, GC, CRC, HCC, and PDAC, are highly heterogeneous and exhibit distinct genetic profiles, stromal structures, microbial environments, and immune contexts. Thus, while some autophagy-related CAF mechanisms, such as metabolic support, cytokine production, and ECM remodeling, may be common across cancers, other autophagy regulatory pathways are likely cancer-type specific. These pathways may be influenced by oncogenic drivers (KRAS, BRAF, TP53, and CDH1 in most of the GI cancers), cancer stage, inflammatory conditions, and microbial or viral infections (e.g., EBV in GC). It is important to recognize this heterogeneity to rationally develop therapeutic approaches targeting autophagy in the tumor stroma.

Progression of GI cancers is influenced by the tumor ME, where CAFs, immune cells, and extracellular matrix interact with a complex network to facilitate invasion, metastasis, and therapy resistance. CAFs, by showing functional and spatial heterogeneity and coordinating stromal remodeling, immunosuppression, angiogenesis, and metabolic reprogramming, create a pro-tumorigenic niche. Autophagy plays a dual role in GI cancers: initially, it prevents cancer by maintaining cellular homeostasis and genomic stability, but later supports tumor growth, CSC renewal, metabolic adaptation, and resistance to therapy. The interplay between CAFs, autophagy, and components and growth factors in the ME creates a complex dynamic in GI malignancies. It highlights therapeutic strategies targeting cancer cells and the supportive ME.

## Data Availability

No datasets were generated or analysed during the current study.

## Abbreviations

CAF: Cancer–associated fibroblast
CEA: Carcinoembryonic antigen
CRC: Colorectal cancer
EMT: Epithelial–to–Mesenchymal Transition
FAP: Fibroblast activation protein
GI: Gastrointestinal
IPMN: Intraductal papillary mucinous neoplasm
MCN: Mucinous cystic neoplasm
MDSC: Myeloid–derived suppressor cell
NK: Natural Killer (cells)
PanIN: Pancreatic Intraepithelial Neoplasia
PD-L1: Programmed Death–Ligand 1
PDAC: Pancreatic Ductal Adenocarcinoma
TAM: Tumor–Associated Macrophage
TME: Tumor Microenvironment
